# Senescence Marker Protein 30 Has a Cardio-Protective Role in Doxorubicin-Induced Cardiac Dysfunction

**DOI:** 10.1371/journal.pone.0079093

**Published:** 2013-12-31

**Authors:** Makiko Miyata, Satoshi Suzuki, Tomofumi Misaka, Tetsuro Shishido, Shu-ichi Saitoh, Akihito Ishigami, Isao Kubota, Yasuchika Takeishi

**Affiliations:** 1 Department of Cardiology and Hematology, Fukushima Medical University, Fukushima, Japan; 2 First Department of Internal Medicine, Yamagata University School of Medicine, Yamagata, Japan; 3 Molecular Regulation of Aging, Tokyo Metropolitan Institute of Gerontology, Tokyo, Japan; Virginia Commonwealth University Medical Center, United States of America

## Abstract

**Background:**

Senescence marker protein 30 (SMP30), which was originally identified as an aging marker protein, is assumed to act as a novel anti-aging factor in the liver, lungs and brain. We hypothesized that SMP30 has cardio-protective function due to its anti-aging and anti-oxidant effects on doxorubicin (DOX)-induced cardiac dysfunction.

**Methods and Results:**

SMP30 knockout (SMP30 KO) mice, SMP30 transgenic (SMP30 TG) mice with cardiac-specific overexpression of SMP30 gene and wild-type (WT) littermate mice at 12–14 weeks of age were given intra-peritoneal injection of DOX (20 mg/kg) or saline. Five days after DOX injection, echocardiography revealed that left ventricular ejection fraction was more severely reduced in the DOX-treated SMP30 KO mice than in the DOX-treated WT mice, but was preserved in the DOX-treated SMP30 TG mice. Generation of reactive oxygen species and oxidative DNA damage in the myocardium were greater in the DOX-treated SMP30 KO mice than in the DOX-treated WT mice, but much less in the SMP30 TG mice. The numbers of deoxynucleotidyltransferase-mediated dUTP nick end-labeling positive nuclei in the myocardium, apoptotic signaling pathways such as caspase-3 activity, Bax/Bcl-2 ratio and phosphorylation activity of c-Jun N-terminal kinase were increased in SMP30 KO mice and decreased in SMP30 TG mice compared with WT mice after DOX injection.

**Conclusions:**

SMP30 has a cardio-protective role by anti-oxidative and anti-apoptotic effects in DOX-induced cardiotoxicity, and can be a new therapeutic target to prevent DOX-induced heart failure.

## Introduction

Anthracyclines are the drugs most closely related to acute and late cardiac toxicity. [1] It has been known since the 1970s that anthracycline treatment is associated with an increased risk of heart failure, and that this is dependent on cumulative dose and schedule. [2], [3] One of the mechanisms responsible for doxorubicin (DOX) cardiotoxicity is the formation of reactive oxygen species (ROS), [4], [5] which can harm membrane lipids and other cellular components, leading to cardiomyocyte apoptosis and death. [6] In addition, oxidative stress is considered to be an important factor of controlling heart aging. [7]

Senescence marker protein 30 (SMP30), a 34-kDa protein, was originally identified as a novel aging marker protein in rat liver, whose expression decreases androgen-independently with age. [8] SMP30 transcripts are detected in almost all organs, and the SMP30 gene is highly conserved among numerous animal species including humans. [9] It has been demonstrated that SMP30 plays multifunctional roles as Ca^2+^ regulator, [10] anti-oxidants, [11] and gluconolactonase which is a key enzyme in the ascorbic acid (vitamin C) biosynthesis. [12] SMP30 knockout (SMP30 KO) mice were generated [13] and showed a shorter life span than that of wild-type (WT) mice on a vitamin C-deficient diet. [14] Using SMP30 KO mice, recent reports have demonstrated that SMP30 functions to protect cells from apoptosis in the liver [13] and that SMP30 has protective effects against age-associated oxidative stress in the brain [15] and lungs [16]. Furthermore, SMP30 KO mice have shown accelerated senescence in the kidney [17] and the worsening of glucose tolerance. [18] Taken together, SMP30 is assumed to behave as an anti-aging factor. Recently, we have demonstrated that deficiency of SMP30 exacerbates angiotensin II-induced cardiac hypertrophy, dysfunction and remodeling in mice. [19]

We hypothesized that SMP30 has cardio-protective functions in response to DOX. To test this hypothesis, we generated transgenic mice with cardiac-specific over expression of SMP30 gene (SMP30 TG). Using SMP30 KO mice and SMP30 TG mice, we examined the effects of SMP30 on DOX-induced cardiac dysfunction *in vivo*.

## Methods

### Ethics statement

The investigations conformed to the *Guide for the Care and Use of Laboratory Animals* published by the US National Institutes of Health (NIH publication, 8th Edition, 2011). Our research protocol was approved by the Fukushima Medical University Animal Research Committee (permit number 21102), and all animal experiments were conducted in accordance with the guidelines of the Fukushima Medical University Animal Research Committee. All efforts were made to minimize suffering animals.

### Animal protocol

SMP30 KO (C57BL/6 background) mice were established as previously reported. [13] We generated SMP30 TG mice (same C57BL/6 background) with cardiac-specific over expression of SMP30 gene using α-myosin heavy chain promoter as previously reported. [20] Briefly, a 5.5 kb fragment of murine α-MHC gene promoter (a kind gift from Dr J. Robbins, Children's Hospital Research Foundation, Cincinnati, OH, USA) and 1.6 kb SMP30 cDNA [9] were subcloned into plasmid. The plasmid was digested with restrictive enzyme to generate a DNA fragment composed of the α-MHC gene promoter, SMP30 cDNA, and a poly A tail of the human growth hormone. We microinjected the construct into the pronuclei of single-cell fertilized mouse embryos to generate TG mice as previously described. [20] Gene and protein expression levels of SMP30 were augmented about ten-fold and five-fold in the SMP30 TG mouse hearts compared with WT littermate mice. WT, SMP30 KO and SMP30 TG mice were fed with regular chow (CLEA Rodent Diet CA-1, CLEA Japan Inc., Tokyo, Japan). Drinking water containing vitamin C (1.5 g/l) was provided for SMP30 KO mice to avoid vitamin C deficiency due to their inability to synthesize vitamin C *in vivo*. We used age-matched (12 to 14 weeks) male WT, SMP30 KO and SMP30 TG mice. Those mice were given intra-peritoneal injection of DOX (20 mg/kg, Sigma Aldrich, St. Louis, MO, USA) or saline.

### Measurement of vitamin C

Total vitamin C levels in the heart were measured by the dinitrophenylhydrazine (DNPH) method according to the manufacturer's protocol (SHIMA Laboratories Co. Ltd., Tokyo, Japan). [21] Briefly, whole heart tissues were homogenized with 5.4% metaphosphoric acid and the supernatant samples were incubated with DNPH at 4°C for 3 hr. After the addition of sulfuric acid, the optical density was measured at 530 nm with a spectrophotometer (n = 5 for each group).

### Echocardiographic measurements

Transthoracic echocardiography was performed at 5 days after DOX or saline injection using a Vevo 2100 High-Resolution In Vivo Imaging System (Visual Sonics Inc., Toronto, Canada) with a high-resolution 40-MHz imaging transducer as previous reports described. [22], [23] The mice were lightly anesthetized by titrating isoflurane (0.5–1.5%) to achieve a heart rate of around 400 beats/min. Parasternal long-axis, short-axis, and apical four-chamber two-dimensional images were acquired. With the use of the M-mode images, left ventricular end-diastolic dimension (LVEDD) and left ventricular end-systolic dimension (LVESD) were measured. The percentage of left ventricular fractional shortening (FS) was calculated as 100×((LVEDD-LVESD)/LVEDD). Left ventricular ejection fraction (EF) was measured using Teichholz formula. To evaluate diastolic function, we measured the ratio of left ventricular inflow E wave to A wave peak velocity (E/A) and the ratio of transmitral early left ventricular filling velocity to early diastolic Doppler tissue imaging of the mitral annulus (E/e′). All measurements were obtained from 3 cardiac cycles and the data were averaged.

### Histopathological analysis

After echocardiography, the mice were sacrificed by cervical dislocation and their hearts were rapidly excised for the analyses. The heart was fixed with 4% paraformaldehide at 4°C overnight and embedded in paraffin. The tissues were cut at the levels of papillary muscle and sectioned into 2-µm thickness. The sections were stained with Elastica-Masson to assess myocardial interstitial fibrosis. The area of fibrosis, which was stained with blue collagen, was determined using Adobe Photoshop CS2 (Adobe Systems Inc., San Jose, CA, USA) and fibrosis fraction was calculated as the ratio of the blue color area to total cardiac area. For this analysis, the digital photomicrographs were taken from 20 random fields at 200× magnification in each section, and the average was obtained from 3 sections.

### Oxidative stress in the heart

The excised heart tissue was immediately frozen in liquid nitrogen with optimal cutting temperature compound and sectioned at 10-µm thickness. [24], [25] The section was incubated with 10 µmol/l dihydroethidium (DHE, Sigma Aldrich Co.) at 37°C for 30 min. The fluorescent images were acquired using fluorescence microscope (Olympus IX71, OLYMPUS Optical Co., Tokyo, Japan), and the mean DHE fluorescence intensity of cardiomyocytes, which were in 20 randomly selected fields in each section, was quantitated with Adobe Photoshop CS2 (Adobe Systems Inc.). [24]

Oxidative DNA damage in the myocardium was evaluated by 8-hydroxy-2′- deoxyguanosine (8-OHdG) immunostaining. [26] Heart tissue sections were stained with anti-8-OHdG monoclonal antibody (clone N45.1, Japan Institute for the Control of Aging, Fukuroi, Japan). Briefly, after deparaffinization, the sections were treated with 0.3% H_2_O_2_ in methanol for 30 min at room temperature, and with 0.1% trypsin for 15 min at 37°C. The sections were then reacted with N45.1 monoclonal antibody (10 µg/ml) for 1 hr at room temperature in a humidity chamber, followed by incubation with DakoEnVision/HRP system (Dako Japan, Tokyo, Japan) for 30 min at room temperature. Sections were then treated with 3, 3′-diaminobenzidine tetrahydrochloride solution (NICHIREI BIOSCIENCES INC., Tokyo, Japan) for 5 min, and counterstaining was carried out with haematoxylin-eosin for 1 min. [27] The positive 8-OHdG nuclei with oxidative DNA damage, which was stained with dark brown, was determined using Adobe Photoshop CS2 (Adobe Systems Inc.), and we calculated the ratio of 8-OHdG positive neclei per total cell number. For this analysis, the digital photomicrographs were taken from 20 random fields at 200× magnification in each section, and the average was obtained from 3 sections.

### Western blotting

Total protein was extracted from the snap-frozen left ventricle using Cell Lysis Buffer (Cell Signaling Technology, Inc., Beverly, MA) with Protease Inhibitor Cocktail (BD Biosciences, San Jose, CA) as previous reports described. [20], [28] Protein concentration was determined by protein assay (DC protein assay kit, Bio-Rad Laboratories, Inc., Hercules, CA, USA). Equal amounts (20 µg) of the protein samples were electrotransferred onto sodium dodecyl sulfate polyacrylamide gel electrophoresis (SDS-PAGE, 5–20%) and transferred onto polyvinylidenedifluoride membranes (ATTO Co., Tokyo, Japan). The primary antibodies used were as follows: anti-Bax, anti-Bcl-2, anti-phospho-stress-activated protein kinase/c-Jun N-terminal kinase (SAPK/JNK, Thr183/Tyr185), anti-SAPK/JNK, anti-phospho-Akt, anti-Akt, anti-phospho-p38 mitogen activated protein kinase (MAPK), and anti-p38 MAPK (Cell Signaling Technology Inc., Danvers, MA, USA). The signals from immunoreactive bands were visualized by an Amersham ECL system (Amersham Pharmacia Biotech UK Ltd., Buckinghamshire, UK) and quantified using the Image J Software (NIH, Bethesda, MD, USA).

### In vivo terminal deoxynucleotidyltransferase-mediated dUTP nick end-labeling assay

Cardiomyocyte apoptosis was detected by the terminal deoxynucleotidyltransferase-mediated dUTP nick end-labeling (TUNEL) method (CardioTACS In Situ Apoptosis Detection Kit, Trevigen, Inc., Gaithersburg, MD, USA). Paraffin-embedded sections (2-µm thickness) of the left ventricle were used and TUNEL staining was performed according to the manufacturer's instructions. The numbers of TUNEL positive nuclei and total nuclei were counted in 3 sections of each sample under light microscopy, and TUNEL positive nuclei were then expressed as a percent of the total nuclei. [28]

### Caspase-3 activity

Caspase-3 activity in myocardial tissues was measured with a CPP32/caspase-3 colorimetric protease assay that recognizes the sequence DEVD. The assay is based on spectrophotometric detection of the chromophore p-nitroaniline (pNA) after cleavage from the labeled substrate DEVD-pNA (CRP32/caspase-3 colorimetric protease kit, BioVision, Milpitas, CA, USA). The pNA light emission can be quantified using a microtiter plate reader at 405 nm.

### Statistical analysis

All data are expressed as mean±standard deviation (SD). Comparisons of vitamin C levels at basal conditions among WT, SMP30 KO and SMP30 TG mice were performed by one-way analysis of variance (ANOVA). All other parameters were evaluated by two-way ANOVA followed by multiple comparisons with Bonferroni test using SPSS Statistics 17.0 (SPSS Japan Inc., Tokyo, Japan). If the data were not distributed normally, the non-parametric test was used. A probability value <0.05 was considered statistically significant.

## Results

### Myocardial vitamin C concentrations

First, we measured vitamin C levels of the heart tissue at basal conditions. To avoid vitamin C deficiency, drinking water containing sufficient vitamin C was supplied for SMP30 KO mice, because SMP30 has a unique gluconolactonase activity in the liver and SMP30 KO mice were unable to synthesize vitamin C due to the lack of gluconolactonase activity. [12] The tissue concentrations of vitamin C level were not significantly different among WT, SMP30 KO and SMP30 TG mice (45.7±7.0, 44.5±10.2, and 46.5±6.6 µg/g tissue, respectively).

### Cardiac function in the DOX- or saline-treated WT, SMP30 KO and SMP30 TG mice

**Figure 1:**
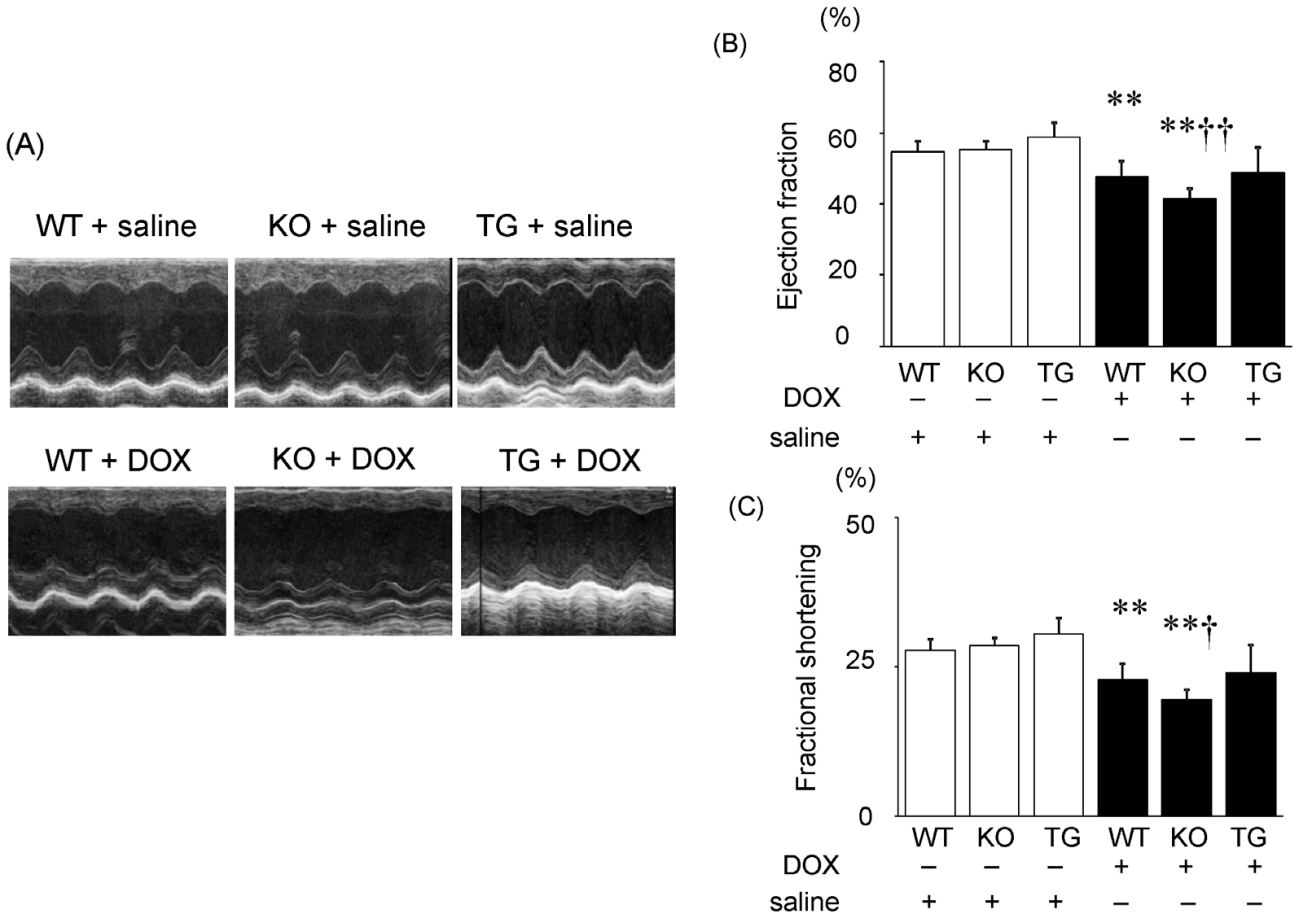
Representative M-mode echocardiograms of WT, SMP30 KO and SMP30 TG mice given saline or DOX. (A) Left ventricular ejection fraction (B) and left ventricular fractional shortening (C) in WT, SMP30 KO and SMP30 TG mice given saline or DOX are shown. Data were obtained from 8 mice in each group. **P<0.01 vs. same genotype mice given saline, †P<0.05 and ††P<0.01 vs. the doxorubicin-treated WT mice.

Echocardiography showed that EF and FS were similar among WT, SMP30 KO, and SMP30 TG mice given saline as shown in Figure 1. However after DOX injection, EF and FS reduced in WT and SMP30 KO mice compared to saline (P<0.01, respectively), but not in SMP30 TG mice (Figure 1). EF and FS were more severely reduced in the DOX-treated SMP30 KO mice compared with the DOX-treated WT mice (P<0.01 and P<0.05, respectively). These echocardiographic data revealed that the left ventricular systolic function was depressed in the DOX-treated SMP30 KO mice compared with the DOX-treated WT mice. In contrast, the left ventricular systolic function was preserved in SMP30 TG mice after DOX. Regarding diastolic function, E/A and E/e′ were not different among the DOX-treated WT mice, the DOX-treated SMP30 KO mice and the DOX-treated SMP30 TG mice (E/A, 1.64±0.28, 2.39±2.76 and 1.84±0.77; E/e′, 36.4±16.7, 20.1±54.6 and 45.2±27.7, respectively).

### Effect of SMP30 deficiency and up-regulation on DOX-induced cardiac fibrosis

**Figure 2:**
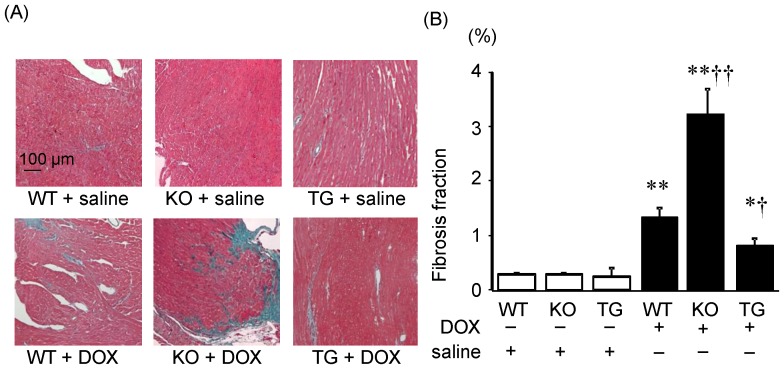
Effect of SMP30 deficiency and up-regulation on DOX-induced cardiac fibrosis. A, Photomicrograph of histological Elastica-Masson stained preparations of heart specimens. B, Fibrosis area/total heart area (%). Data were obtained from 7 mice in each group. *P<0.05 and **P<0.01 vs. same genotype mice given saline, †P<0.05 and ††P<0.01 vs. the doxorubicin-treated WT mice.

The degree of cardiac fibrosis was significantly higher in the DOX-treated SMP30 KO mice and lower in the DOX-treated SMP30 TG mice compared to the DOX-treated WT mice (P<0.01 and P<0.05, respectively) as shown in Figure 2. These data revealed that the deficiency of SMP30 exacerbated DOX-induced cardiac fibrosis. On the contrary, up-regulated SMP30 inhibited DOX-induced cardiac fibrosis.

### Effect of SMP30 on DOX-induced myocardial oxidative stress

**Figure 3:**
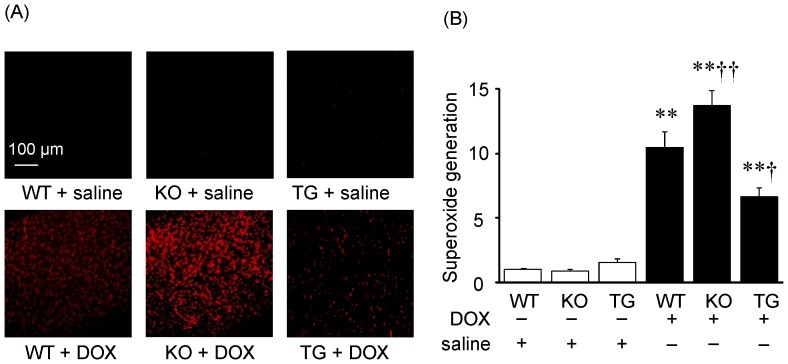
DOX-induced myocardial oxidative stress in WT, SMP30 KO and SMP30 TG mice. A, Photomicrograph of DOX-induced superoxide formation from frozen heart sections using dihydroethidium (DHE). B, The mean of DHE fluorescence intensity of cardiomyocytes. Data were obtained from 8 mice in each group. **P<0.01 vs. same genotype mice given saline, †P<0.05 and ††P<0.01 vs. the doxorubicin-infused WT mice.

Several reports revealed that SMP30 has protective effects against oxidative stress in organs other than the heart, [14], [15] therefore we evaluated the generation of ROS in heart tissue by DHE staining, which indicates the super oxide levels of living cells (Figure 3A). The ROS generation in the DOX-treated SMP30 KO mice was greater than that in the DOX-treated WT mice (P<0.01). In contrast, the generation of ROS in the DOX-treated SMP30 TG mice was smaller than that in the DOX-treated WT mice (P<0.05) as shown in Figure 3B.

**Figure 4:**
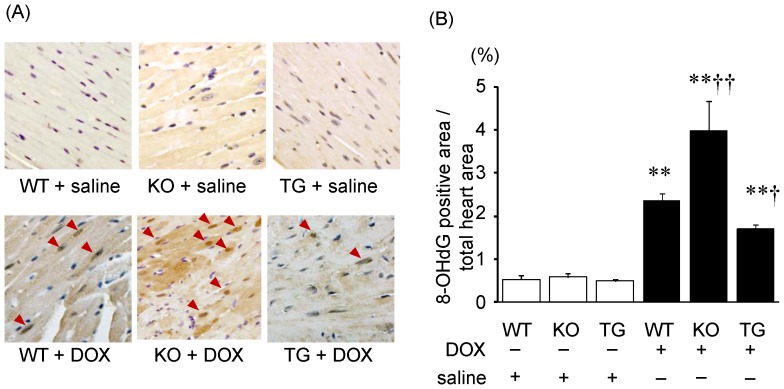
Immunohistochemical detection of oxidative DNA damage with 8-OHdG. A, Photomicrographs of the 8-OHdG positive nuclei, which were stained with dark brown, prepared from heart specimens. B, 8-OHdG positive nuclei/total cells (%). Data were obtained from 7 mice in each group. **P<0.01 vs. same genotype mice given saline, †P<0.05 and ††P<0.01 vs. the doxorubicin-infused WT mice.

Oxidative DNA damage in the myocardium was evaluated by 8-OHdG immunostaining. After DOX injection, 8-OHdG positive nuclei were observed in cardiomyocytes (Figure 4A), and a ratio of 8-OHdG positive nuclei/total cells was significantly increased in SMP30 KO mice compared with WT mice (P<0.01), but less in SMP30 TG mice (P<0.05, Figure 4B). These data suggested that deficiency of SMP30 exacerbated and overexpression of SMP30 inhibited DOX-induced myocardial oxidative stress.

### Effect of SMP30 deficiency and up-regulation on DOX-induced cardiac cell apoptosis

**Figure 5:**
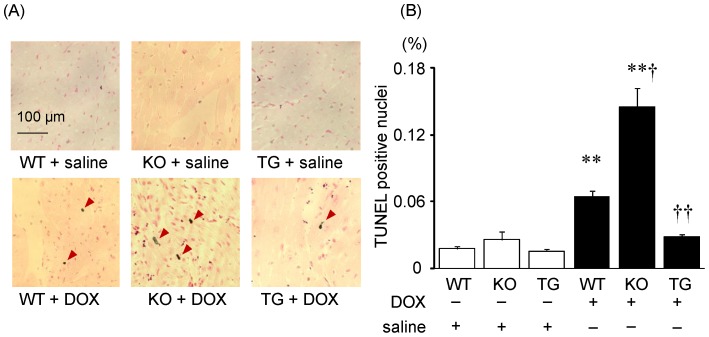
Comparisons of cardiac cell apoptosis between WT, SMP30 KO and SMP30 TG mice. A, Representative TUNEL stained sections from indicated groups. Arrows indicate TUNEL positive nuclei. B, TUNEL positive nuclei were expressed as a percent of the total nuclei. Data were obtained from 7 mice in each group. **P<0.01 vs. same genotype mice given saline, †P<0.05 and ††P<0.01 vs. the doxorubicin-infused WT mice.

As a previous report demonstrated, SMP30 also has anti-apoptotic effects. [12] We therefore checked cardiac cell apoptosis using TUNEL staining (Figure 5A). TUNEL-positive nuclei were few in number in control WT, SMP30 KO and SMP30 TG mice given saline. After DOX injection, the percentage of TUNEL-positive nuclei was higher in SMP30 KO mice and lower in SMP30 TG mice compared to that in WT mice as shown in Figure 5B (P<0.05 and P<0.01, respectively).

**Figure 6:**
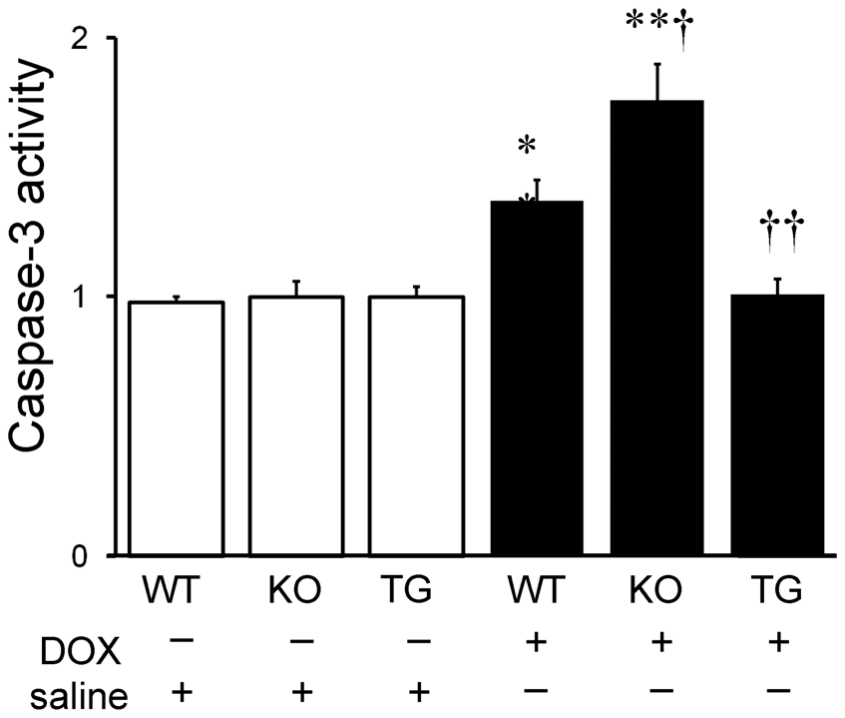
Caspase-3 activity in WT, SMP30 KO and SMP30 TG mice after DOX or saline. Data were obtained from 7 mice in each group. **P<0.01 vs. same genotype mice given saline, †P<0.05 and ††P<0.01 vs. the doxorubicin-infused WT mice.

**Figure 7:**
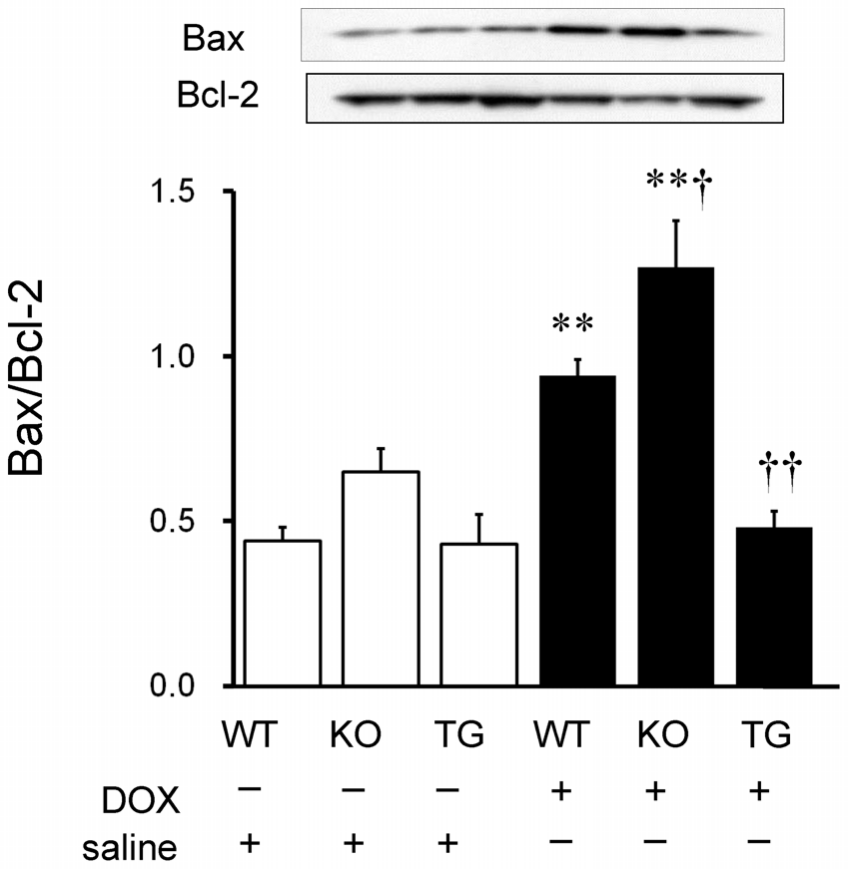
Protein expressions of Bax and Bcl-2 in the myocardium of WT, SMP30 KO and SMP30 TG mice after saline or DOX infusion. The ratios of Bax to Bcl-2 obtained from 7 mice in each group are shown in the bar graph. **P<0.01 vs. same genotype mice given saline, †P<0.05 and ††P<0.01 vs. the doxorubicin-infused WT mice.

**Figure 8:**
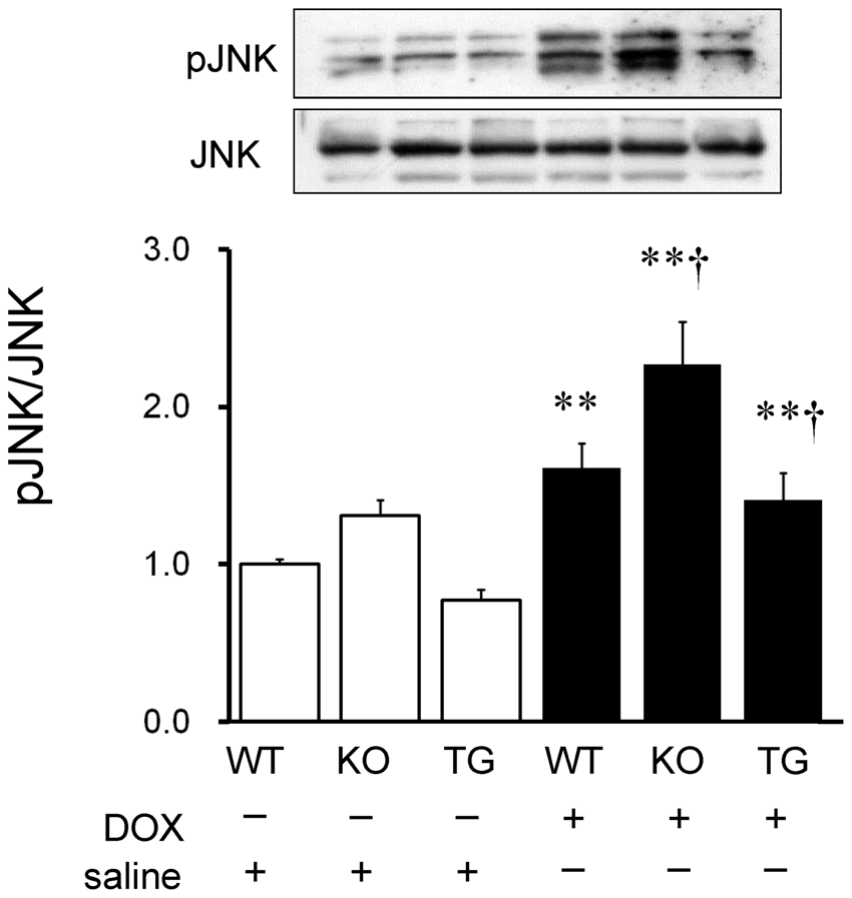
Phosphorylation activity of SAPK/JNK in WT, SMP30 KO and SMP30 TG mice heart tissue after saline or DOX infusion. The ratios of phosphorylated SAPK/JNK (Thr183/Tyr185, pJNK) to total SAPK/JNK (JNK) obtained from 7 mice in each group are shown in the bar graph. **P<0.01 vs. same genotype mice given saline, †P<0.05 vs. the doxorubicin-infused WT mice.

We then examined signaling pathways of DOX-induced apoptosis in the heart. Caspase-3 is a key mediator of apoptosis, and activation of caspase-3 leads to DNA injury and subsequently apoptotic cell death. [29] The activation of caspase-3 was induced by DOX infusion in WT and SMP30 KO mice (P<0.01, respectively), but not in SMP30 TG mice (Figure 6). Caspase-3 activity was higher in the DOX-treated SMP30 KO mice and lower in the DOX-treated SMP30 TG mice compared with the DOX-treated WT mice (P<0.05 and P<0.01, respectively) as demonstrated in Figure 6. Western blot analysis showed that Bax expression, which functions as pro-apoptotic protein, was increased in WT and SMP30 KO mice, but not in SMP30 TG mice, after DOX (Figure 7). In contrast, the expression of anti-apoptotic protein Bcl-2 was decreased in WT and SMP30 KO mice, but not in SMP30 TG mice, after DOX. The ratio of Bax to Bcl-2 was higher in the DOX-treated SMP30 KO mice and lower in the DOX-treated SMP30 TG mice than in the DOX-treated WT mice (P<0.05 and P<0.01, respectively). Next, we examined the involvement of SAPK/JNK, which has a crucial role in cell apoptosis as a subgroup of the mitogen-activated protein kinase family. [30] Phosphorylation activity of SAPK/JNK was increased in the DOX-treated SMP30 KO mice and was decreased in the DOX-treated SMP30 TG mice compared with the DOX-treated WT mice (P<0.05, respectively) as shown in Figure 8. These findings demonstrated that deficiency of SMP30 exacerbated DOX-treated cardiac cell apoptosis through the activation of these signaling pathways, and overexpression of SMP30 blocked DOX-induced apoptotic signaling. Phosphorylation activity of Akt was not changed among the DOX-treated WT mice, the DOX-treated SMP30 KO mice and the DOX-treated SMP30 TG mice (0.88±0.04, 0.80±0.09 and 0.53±0.11, respectively). Similarly, phosphorylation activity of p38 MAP kinase showed no significant differences among the DOX-treated WT mice, the DOX-treated SMP30 KO mice and the DOX-treated SMP30 TG mice (0.79±0.08, 0.62±0.50 and 0.59±0.06, respectively).

## Discussion

In this study, we demonstrated the first evidence using the loss and gain of functional approach with genetically engineered mice that SMP30 has a cardio-protective role with anti-oxidative and anti-apoptotic effects in the DOX-treated hearts.

### Doxorubicin cardiotoxicity

Doxorubicin has been used as a potent anticancer agent, but serious dose-dependency cardiotoxicity precludes its use in a wide range of patients. [2], [31] Cardiotoxicity is a major limiting factor in anti-cancer therapy using doxorubicin. [31]–[33] In addition to the blockade of tumor cell growth, doxorubicin generates ROS, which directly damage cellular components in various types of cells including cardiomyocytes. [2], [31] Accumulated data suggest that cardiomyocyte apoptosis and death in response to doxorubicin are mainly mediated by increased oxidative stress. [2], [34]

Interestingly, we found that the DOX-treated SMP30 KO mice showed depressed left ventricular systolic function compared with the DOX-treated WT mice, suggesting the absence of SMP30 caused more progressive cardiac dysfunction (Figure 1). Moreover, we observed that deficiency of SMP30 exacerbates DOX-induced cardiac fibrosis in SMP30 KO mice, and DOX-induced cardiac fibrosis was inhibited in SMP30 TG mice (Figure 2). The SMP30 KO mice had much more generated ROS by DOX stimulation (Figure 3). In addition, the SMP30 KO mice were far more susceptible to DOX-induced apoptosis associated with activation of caspase-3, increase of Bax, decrease of Bcl-2 and phosphorylation of SAPK/JNK. In the SMP30 TG mice, activation of caspase-3 and Bax/Bcl-2 ratio were attenuated, and phosphorylation of SAPK/JNK was decreased to the contrary (Figures 6, 7, and 8). These data indicate that SMP30 has a protective role against DOX-associated cardiac dysfunction by inhibiting oxidative stress and cardiac cell apoptosis.

### Functional role of SMP30 in the heart

The known factors involved in senescence of cardiomyocytes include oxidative stress, altered gene expressions, inflammation, reduced cellular protection and repair, altered cellular metabolism, altered protein degradation machinery and autophagy machinery, among others. [35] SMP30 has been proposed as an important aging marker, and the lack of SMP30 causes various dysfunctions of organs during aging process. SMP30 KO mice were highly susceptible to tumor necrosis factor-α and Fas-mediated apoptosis. [13] Oxidative stress was increased in the brains of SMP30 KO mice without influencing antioxidant enzyme status. [15] SMP30 KO mice also showed senile lung like pulmonary emphysema, and SMP30 protected the lung from oxidative stress associated with aging and smoking. [16] Accelerated senescence of renal tubular epithelial cells was observed in SMP30 KO mice. [17] Moreover, the potent anti-aging and anti-oxidative actions of a low-calorie diet effectively suppressed the age-related downregulation of SMP30 by ROS reduction, indicating that SMP30 expression was influenced by oxidative stress. [36]

### Study limitations and clinical implications

Although we demonstrated in this study that the systemic loss of SMP30 exacerbates DOX-induced cardiac dysfunction, cardiac remodeling is modified by functions of extra-cardiac organs such as the kidney. Our present data may not necessarily be the result from cardiac specific protective effects of SMP30 on the heart. Further studies using the animal model of cardiac specific deletion of SMP30 are required.

In the present study, we demonstrated that cardiac specific overexpression of SMP30 gene attenuated DOX-induced superoxide generation, oxidative DNA damage, activation of SAPK/JNK and caspase-3, increases in Bax/Bcl-2 ratio, and cardiac cell apoptosis. Consequently, cardiac function was preserved after DOX in SMP30 TG mice. Therefore, SMP30 will be a novel therapeutic target for DOX-induced cardiotoxicity and subsequent heart failure.

### Conclusions

The results of this study demonstrated that DOX-induced cardiotoxicity is aggravated in SMP30 KO mice by the exacerbating of superoxide generation, leading to enhanced apoptosis of cardiac cells. In contrast, overexpression of SMP30 in the heart prevented these processes in response to DOX. SMP30 has a cardio-protective role by anti-apoptotic and anti-oxidative effects in DOX-induced cardiotoxicity.
